# Development and evaluation of the Digit Triplet Test in Swahili language

**DOI:** 10.4102/sajcd.v72i1.1090

**Published:** 2025-02-21

**Authors:** Bjørn G. Rosendahl, Tron V. Tronstad, Jon Øygarden

**Affiliations:** 1 Department of Ear, Nose and Throat, Head and Neck Surgery, Lovisenberg Diaconal Hospital, Oslo, Norway; 2 Department of Special Needs Education, Faculty of Educational Sciences, University of Oslo, Oslo, Norway; 3 Department of Sustainable Communication Technologies, SINTEF Digital, Trondheim, Norway; 4 Department of Electronic Systems, Information Technology and Electrical Engineering, The Norwegian University of Science and Technology, Trondheim Norway

**Keywords:** Digit Triplet Test, Swahili, hearing screening, speech reception threshold, speech in noise, language-specific hearing test

## Abstract

**Background:**

The development of a Digit Triplet Test in the Swahili language is an essential step towards providing accurate hearing assessment for Swahili-speaking populations.

**Objectives:**

This study aimed to develop a Digit Triplet Test in Swahili through a two-part procedure consisting of an optimisation phase and an evaluation phase using normal hearing participants.

**Method:**

A total of 34 subjects participated in the study. During the optimisation phase, a psychometric intelligibility function was determined for each recorded digit, allowing for volume adjustments to standardise the threshold across all digits. This resulted in a lower threshold and a steeper psychometric function for both the triplets and the test lists. Using the optimised speech material, four test lists were created, each containing 27 triplets composed of digits between 1 and 9. The finalised material was then evaluated.

**Results:**

In the final version, the mean Speech Reception Threshold (SRT) for the participants was −8.9 ± 0.6 dB Signal-to-Noise Ratio (SNR), and the mean slope was 24.7 ± 3.5%/dB using triplet scoring.

**Conclusion:**

The psychometric function for normal listeners shows a steep slope with little variation between subjects and across test lists.

**Contribution:**

The test results are comparable to those of Digit Triplet Tests developed in other languages, indicating the effectiveness and reliability of the Swahili Digit Triplet Test for hearing assessments.

## Introduction

Difficulties in hearing speech in noise are well-known challenges faced by individuals with hearing loss (Houtgast & Festen, 2008). Speech-in-noise tests have therefore proven to be valuable audiological tools for screening, evaluation, diagnosis, and scientific research. These tests are particularly well-suited for screening for several reasons. Firstly, the stimuli are presented at suprathreshold sound levels, which reduce the demands on the testing environment. Secondly, the speech recognition threshold (SRT) (the signal-to-noise ratio [SNR] that yields 50% speech intelligibility) is not affected by the absolute presentation level over a wide range of intensities (Wagener & Brand, 2005), making calibration of the screening equipment less critical. In addition, the test does not rely on exact speech transmission because both the noise and the speech signal are filtered by the same transducer (Zokoll et al., 2012).

These features were utilised by Smits et al. (2004) in developing a telephone-based screening test, the Digit Triplet Test (DTT), where digit triplets (e.g. 2-5-1) are presented in speech-shaped noise with the same long-term average spectrum as the digits. The test subject responds by entering the numbers they perceive on the telephone keypad. The speech material produces a steep intelligibility function, resulting in SRTs with low standard deviation (Smits et al., 2004).

Subsequent initiatives have aimed to standardise this test, facilitating data comparison across countries. In 2012, a German version was proposed as an optimisation prototype (Zokoll et al., 2012), and in 2015, the International Collegium of Rehabilitative Audiology (ICRA) published recommendations for constructing multilingual speech tests (Akeroyd et al., 2015). Today, the DTT exists in several languages, some developed within the HearCom project (Vlaming et al., 2011) with funding from the European Union.

Because of the simplicity of the test and its use of a closed set of speech material, it can easily be implemented as an automated, adaptive, self-administered test on a tablet or online (Willberg et al., 2016). The test can be performed either by using the phone keypad or by displaying the numbers on a smartphone or tablet screen, making remote screening possible.

The Swahili version of the test was developed as part of the ‘I Hear You’ project, which aims to ensure equitable access to inclusive education and participation for children with hearing loss. This article details the selection, recording, and processing of the speech material, the subsequent optimisation procedure to standardise the word-specific SRT, and the evaluation procedure to ensure list equivalence and establish normative data.

## Research methods and design

### Recording and processing of the speech material

A female speaker was chosen for the recording of the speech material as she represents an ‘acoustic compromise’ between male and children’s speech (Akeroyd et al., 2015). The speaker was an adult from Dar-es-Salaam who grew up with Swahili as her first language and had no formal training as a speaker.

The recording took place in a small room with light acoustic treatment, typically used for music recording. The room’s reverberation time was less than 0.3 s between 250 Hz and 8000 Hz, and the setup met the ISO 8253-3 (2012) requirements for recording speech material for speech tests. The material was recorded digitally using Avid Pro Tools 12 through an Apogee Symphony MK1 sound interface in 24 bit and 48 kHz WAV format. The microphone, a Neumann KM 84, was placed 25 cm from the mouth at the same height and at an azimuth of 45 degrees. This placement, adapted from Byrne et al. (1994), was used to avoid air pressure from plosives hitting the microphone membrane.

The digits 0 to 9 were recorded in triplets, with each digit occurring in all three positions within the triplet, a technique adapted from Wagener and Brand (2005). The speech rate was maintained at approximately 140 syllables per minute. The material was recorded seven times, and the most naturally pronounced version of each digit, free from acoustic artefacts, was selected. All recorded material was filtered with a high-pass 18 dB/octave FIR filter at 50 Hz to remove unwanted low-frequency noise. To ensure all digits had the same number of syllables, the digit 0 (‘sifuri’) was excluded. The remaining numbers 1 to 9 each have two syllables in Swahili.

### Cutting the speech material and re-synthesising the triplets

At this stage, intensity differences between the digits were adjusted to provide a better basis for the subsequent optimisation procedure. Notably, the last pronounced digits of each recorded triplet were quieter than the first two digits. This decrease in speech intensity as the sentence progresses is a common phenomenon in normal speech (Versfeld et al., 2000), but uneven levels between digits can result in a less steep psychometric function (Zokoll et al., 2015). Because of the short duration of digits, Root Mean Square (RMS) and other loudness normalisation methods are of limited use. Smits et al. (2004) linearly increased the volume of the recorded triplets by 6.0 dB from the first to the last triplet, including the pauses between and after the digits, resulting in a 3.5 dB difference in intensity between the first and last digit. In this study, the adjustment was done through subjective listening by a professionally trained sound recording engineer. The digits were split into single digits and played back monaurally through a pair of TDH-39 headphones along with the masking noise (described in the next paragraph) set at 65 dBA. Each digit was level-adjusted separately.

Following the ICRA suggestion (Akeroyd et al., 2015), a spacing of 160 ms was added after each digit. Additionally, as performed by Willberg et al. (2016), digits that start with plosives were cut 10 ms to 20 ms before the beginning of the sound, while all other digits were cut as close to the beginning as possible. The digits were then reassembled into 8 test lists, each consisting of 27 digits to be used in the optimisation procedure. Each test list included all three versions (in all three positions of the triplet) of the digits 1 to 9, occurring three times throughout the list. The numbers were organised in a pseudo-random order, ensuring no number appeared more than once in any triplet.

### Development of the masking noise

The quasi-stationary masking noise was created using all digits from the selected speech material to ensure a long-term average spectrum identical to that of the triplets. The speech material was superimposed 10 000-fold to prevent fluctuations in the noise, with both random initial delays and delays between each digit.

### Participants

A total of 34 adults participated in the two phases of the development. Eleven participants, aged 23 years to 47 years (30.8 ± 6.9 years), took part in the optimisation procedure, and 23 participants, aged 16 years to 49 years (33.3 ± 7.3 years), took part in the evaluation procedure. All participants considered Swahili to be their first language, although some grew up speaking both Swahili and a local tribal language or their country’s official language. Twenty-five participants grew up in Tanzania, eight in Kenya, one in Uganda, one in Norway, and one in Canada. All participants were tested for normal hearing, with pure-tone thresholds of 20 dB hearing level (HL) or better at octave frequencies from 125 Hz to 8000 Hz, including 6000 Hz. The testing was conducted in a sound-attenuated booth using an Aurical Aud audiometer with TDH-39 headphones.

The optimisation and evaluation procedures took place at Lovisenberg Diaconal Hospital in Oslo and at the Norwegian University of Science and Technology (NTNU) in Trondheim’s audiology lab, in rooms typically used for audiological tests and counselling.

### Optimisation

In the optimisation phase, intelligibility functions are to be established for all digits in each position of the triplet. Following the ICRA recommendations (Akeroyd et al., 2015) of using at least 10 normally hearing, native-language subjects, 11 subjects participated in this phase. The testing was conducted in a sound-attenuated booth. Audio playback was managed through an Apogee Symphony MK1 sound interface and TDH-39 headphones, connected to a computer running Avid Pro Tools 12 audio editing software. The noise level was calibrated to *L_p,A,eq_* = 65 dB using a Brüel and Kjær 2270 sound level meter and a Larson Davis AEC100 6cc coupler equipped with a Larson Davis Model 2575 microphone. The sound was presented monaurally to the participant’s better ear, and participants were asked to type the numbers they perceived on a numeric USB keypad. After completing a practice list at −1.5 dB SNR, each participant completed 7 lists at 7 different SNRs: −3.5 dB, −5.5 dB, −7.5 dB, −9.5 dB, −11.5 dB, −13.5 dB and −15.5 dB.

By matching the intelligibility function for each digit as closely as possible, the slope of the discrimination function for the test list increases (Akeroyd et al., 2015). In this study, the logistic model function adapted from Zokoll et al. (2012) was used to determine the discrimination function. The probability of answering correctly can be expressed as Equation 1:

$$p(L,L_{50},s_{50})=\frac{1}{1+e^{4.s_{50}(L_{50}-L)}},$$ [Eqn 1]

where *L* is the SNR for the digit, *L*_50_ is the SRT, and *s*_50_ is the slope at this threshold. Chance level and lapse of attention were not included in this study.

A maximum likelihood estimator formula adapted from Brand and Kollmeier (2002) was used to estimate the SRT and the slope. The likelihood function used was Equation 2:

$$l\left(p\left(L,L_{50},s_{50}\right)\right)={\prod_{k=1}^{m}p\left(L_{k},L_{50},s_{50}\right)}^{c(k)}.{\left[1-p\left(L_{k},L_{50},s_{50}\right)\right]}^{1-c(k)},$$ [Eqn 2]

where *c*(*k*) = 1 if the word *k* is repeated correctly and *c*(*k*) = 0 if not. The maximum likelihood discrimination function is found by varying *L*_50_ and *s*_50_ and maximising the logarithm of the likelihood function, log (*l*(*p*(*L,L*_50_,*s*_50_))).

The digits were then adjusted in volume to match at the 80% scoring point, which is estimated to result in a 50% SRT score for the triplet (Smits & Houtgast, 2006).

### Evaluation measurements

With the volume-adjusted digits, four lists were created, each consisting of 27 triplets. For the evaluation phase, data collected from 23 participants were used to establish the psychometric discrimination function for the normative data and to ensure test list equivalence. The setup was identical to that used in the optimisation phase. The noise was played back at *L_p,A,eq_* = 65 dB, and the better ear was chosen as the test ear. Each participant completed four different lists at three different SNRs, estimated to yield scores of 80%, 50% and 20% using triplet-specific scoring (i.e. all three digits had to be perceived correctly in the correct order). The SNRs were −7 dB, −8.5 dB and −10 dB, respectively.

Participants performed a training list consisting of 20 triplets at −4.5 dB SNR before data collection began. The lists were selected in a pseudo-random order, but the first list was always played back at −7.0 dB SNR. Participants could take short breaks as needed. Most took one break, while some needed two. All 12 lists took about 40 min to complete, excluding breaks. Discrimination functions were fitted for all four test lists, participants, and digits.

A one-way analysis of variance was performed using the SRT data to investigate test list equality.

### Ethical considerations

The study was approved by the Norwegian Centre for Research Data (ref. 58283) and the National Institute for Medical Research (NIMR) in Tanzania (NIMR/ HQ/R.8a/ Vol.IX/3009). All procedures performed in studies involving human participants were in accordance with the ethical standards of the institutional and national research committee and with the 1964 Helsinki Declaration and its later amendments or comparable ethical standards. Written informed consent was obtained from all individual participants involved in the study. Participants did not receive any salary but were offered a gift card worth 250 NOK after the testing as a token of appreciation for their time and effort.

## Results

### Results from the optimisation measurements

**FIGURE 1:**
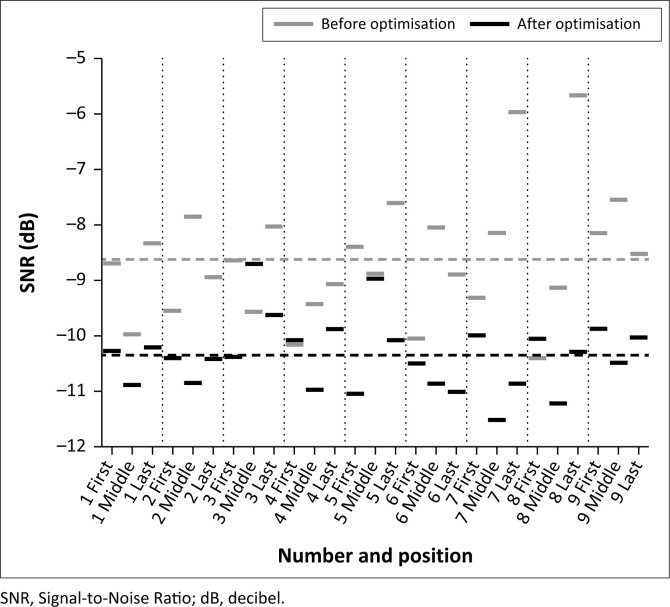
In light grey: The digit specific (all three positions of the triplet) thresholds for 11 subjects that participated in the optimisation phase. The mean threshold for all digits is shown as a dashed line. In black: The digit specific (all three positions of the triplet) thresholds for 23 subjects evaluating the test after optimisation. The mean threshold for all digits is shown as a dashed line.

The results from the first round of testing showed a mean SRT for all digits of -8.6 ± 1.1 dB with mean slope value of 25.0 ± 6.8%/dB. The digit-specific thresholds from the optimisation phase are shown in light grey in Figure 1.

To match the predicted 80% scoring point, the average digit was shifted by 0.9 ± 0.9 dB. Only two digits were shifted by more than 1.8 dB (3.0 dB and 3.2 dB, respectively). Compared to the raw recording, this resulted in level adjustments ranging from ± 3.7 dB, except for the three digits 5, 8 and 7 (all in the last position), which were shifted by 4.8 dB, 6.0 dB and 11.2 dB, respectively.

### Results from the evaluation measurements

The psychometric functions were found to be steep with little variation between the test lists. The main findings from the evaluation are presented in Table 1.

**TABLE 1:** Main findings from the evaluation measurements.

Measurement type	Digit scoring	Triplet scoring
Subject SRT (mean)	−10.3 ± 0.6 dB SNR	−8.9 ± 0.6 dB SNR
Subject slope (mean)	21.7 ± 5.4%/dB	24.7 ± 3.5%/dB
Test list SRT (mean)	−10.3 ± 0.2 dB SNR	−8.9 ± 0.1 dB SNR
Test list slope (mean)	20.0 ± 1.2%/dB	22.9 ± 0.8%/dB

SRT, Speech Reception Threshold; SNR, Signal-to-Noise Ratio; dB, decibel.

**FIGURE 2:**
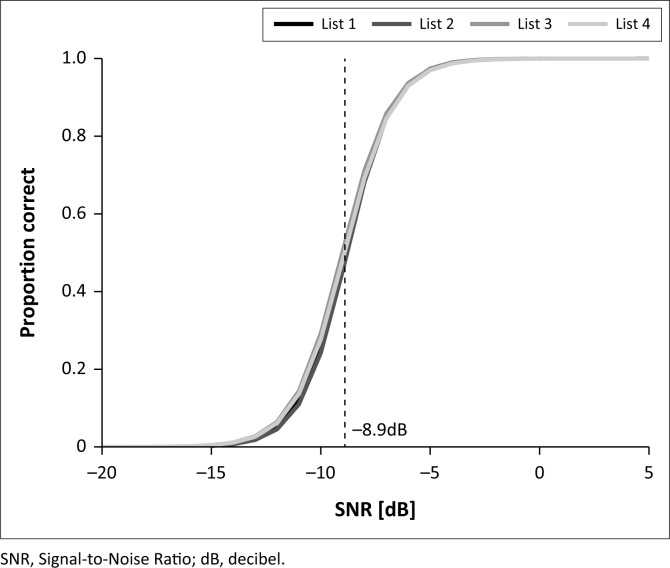
Psychometric functions for the four test lists using triplet scoring. Dashed line shows mean thresholds for all four test lists.

The mean threshold for the four test lists with triplet scoring was −8.9 ± 0.1 dB SNR, and the mean slope was 22.0 ± 0.8%/dB. The psychometric functions for the four test lists using triplet scoring are shown in Figure 2. The mean threshold for the four test lists using digit scoring was −10.3 ± 0.2 dB SNR, with a slope of 20.0 ± 1.2%/dB. A one-way analysis of variance showed no statistically significant difference in SRT between the test lists (*F*(3,88) = 0.66, *p* = 0.58).

**FIGURE 3:**
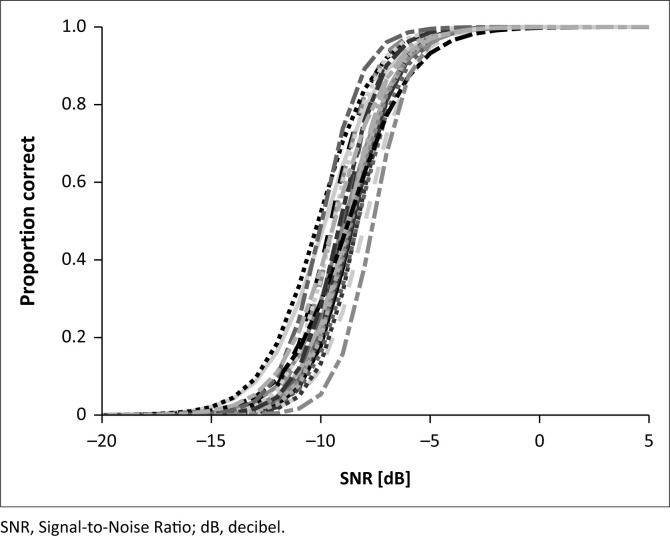
Psychometric functions for 23 subjects using triplet scoring.

The psychometric discrimination function and the SRT, proposed as a normative dataset, were determined by averaging the results from the 23 participants in the evaluation phase. The mean SRT using triplet scoring was −8.9 ± 0.6 dB SNR, and the mean slope was 24.7 ± 3.5%/dB. The psychometric functions for all 23 participants using triplet scoring are shown in Figure 3.

The mean SRT for the single digits was −10.3 ± 0.6 dB SNR, with a mean slope of 21.7 ± 5.4%/dB. The digit-specific thresholds from the evaluation phase are shown in black in Figure 1.

## Discussion

In this study, the Swahili language version of the DTT was developed, optimised, and evaluated. With a mean SRT of -8.9 ± 0.6 dB and a mean slope of 24.7 ± 3.5%/dB at the threshold, the Swahili DTT shows comparable results to DTTs developed for broadband headphones in other languages. Many of these tests were developed within the HearCom project, where the SRTs range from −6.9 dB SNR (Swedish) to −12.7 dB SNR (Russian), and the slopes range from 14.8%/dB (Russian) to 27.1%/dB (French) (Vlaming et al., 2011; Zokoll et al., 2013). Among the languages in which triplet scoring and standard deviation are available, the German DTT (Zokoll et al. 2012) has a threshold of –9.3 ± 0.2 dB SNR and a slope of 19.6 ± 2.2%/dB, the Finnish DTT (Willberg et al., 2016) has a threshold of –10.8 ± 0.5 dB SNR and a slope of 23.4 ± 5.2%/dB and the French DTT (Jansen et al., 2010) has a threshold of –6.4 ± 0.4 dB SNR and a slope of 17.1 ± 2.5%/dB.

The normative data presented in this article are based on measurements from 23 normally-hearing subjects, which approximates the recommended number of normal hearing subjects (25) for normative reference curves stated in the ISO 8253-3 (2012). The group size is also comparable with that of similar studies. The normative value of this study reflects the composition of the participants’ various ages and Swahili dialects, and one should expect variations between different age groups and groups of different dialects.

The psychometric function used in the evaluation does not account for lapses of attention or chance levels. Any effect of this would be most prominent at the lower and upper ends of the function. Therefore, it is assumed not to significantly affect the results, as participants were tested at SNRs in the middle of the psychometric function, where it was estimated that participants would answer between 20% and 80% correctly. Averaging the results from 23 participants further reduces the possible effect of these outliers.

Both the optimisation and evaluation phases of this version of the DTT were conducted using TDH-39 headphones. Jansen et al. (2010) found that when their version of the DTT in French was played back through both broadband headphones and a reduced bandwidth telephone line, the threshold for the number ‘six’ diverged significantly between the two transducers compared to the other numbers. This divergence was attributed to the spectral content of the word, with most energy located above 3000 Hz, whereas a typical telephone line limits the audio frequency range to between 300 Hz and 3400 Hz. In Swahili, the numbers 6, 7, and 9 (sita, saba, tisa) all contain spectral content above 3000 Hz, and a similar divergence between telephone line and broadband headphones is likely to occur with the Swahili version. Potgieter et al. (2016) evaluated their version of the DTT in South African English on five different broadband headphones and found no statistical differences between the average SRTs. Their version was optimised using Sennheiser HD202II and three consumer intra-concha headphones, along with a pair of TDH50P audiometric headphones. If this Swahili version of the DTT is to be used as a self-screening tool with the test subjects’ headphones, further investigation is needed to study the effect of different transducers.

This study focused solely on normal hearing participants to establish normative data. Future studies should include individuals with varying degrees of hearing loss to further strengthen the clinical application of the test. Further improvements to the test could include using different noise signals or incorporating antiphasic speech signals. Such speech signals have been shown to improve the clinical sensitivity to detect sensorineural and asymmetrical hearing loss (De Sousa et al., 2020).

At present, the test material is not incorporated in any test application or smartphone app such as the World Health Organization’s hearWHO or similar, but this will be an appropriate next step to improve accessibility and scalability and is encouraged by the authors.

## Conclusion

This article describes the development of the DTT in Swahili following an optimisation and evaluation procedure. The psychometric function for normal listeners shows a steep curve of 24.7 ± 3.5%/dB with an average SRT of –8.9 ± 0.6 dB SNR. The results show small variation between subjects and across test lists, and the test shows comparable results with DTTs developed for other languages.
